# Injuries to Nerves and Their Consequences

**Published:** 1872-05

**Authors:** 


*Injuries to Nerves and their consequences.* By S. WEIR MITCHELL, M. D. Philadelphia: J. B. LIPPINCOTT & Co. 1872.

The above mentioned work comprises a volume of over 370 pages on a subject, which although it has for some time been considered as of minor importance is coming to be more investigated more thoroughly understood, and hence, more scientificially treated. In the fourteen chapters into which this work is devided, Dr. Mitchell treats of the Anatomy of Nerves, Neuro-Physiology, Physiological Pathology, Varieties of mechanical, and Symptomology of Nerve Lesions, Remote Symptoms, Sensory Lesions, Diagnosis and Prognosis of Injuries to Nerves, Treatment, Lesions of special Nerves, and Neural maladies of stumps.

Each of these subjects are treated clearly and to sufficient length to make the subject understood. In a large portion of the work the subjects are illustrated by cases, many of which are taken from those seen by the Author during tne late war.

On Page 291 in a table of cases of operations for relief of traumatic neuralgia, the author misquotes a case of excision of three inches of the median nerve performed by us in 1868 and reported in this Journal for June of that year. The result as stated by the author is "paralysis of extensors and loss of sensation in back of fore-arm" while the real result was that sensation and motion are perfect. The case was undoubtedly confounded with one of exsection of the muculo-spiral nerve reported in the same article.

Questions involving inquiries to nerves are constantly coming up for consideration and the treatise of Dr. Mitchell will no doubt be referred to with profit by those practitioners who may be so fortunate as to possess a copy. His work constitutes a practical contribution to the subject of injuries to nerves, which will be appreciated by all who have had experience in the diagnosis and treatment of these accidents.

